# Editorial: Physical Activity: Epigenetic and Metabolic Regulation of Brain Aging

**DOI:** 10.3389/fnagi.2020.00195

**Published:** 2020-07-17

**Authors:** Simona Daniele, Chiara Giacomelli, Paul D. Loprinzi, Ferdinando Franzoni

**Affiliations:** ^1^Department of Pharmacy, University of Pisa, Pisa, Italy; ^2^Department of Health, Exercise Science and Recreation Management, University of Mississippi, Oxford, MS, United States; ^3^Department of Clinical and Experimental Medicine, University of Pisa, Pisa, Italy

**Keywords:** physical exercise, brain metabolism, brain aging, epigenetic modifications, neurodegenerative disorders

Physical activity (PA) exerts several benefits in the prevention and delay of age-related cognitive decline and the subsequent development of age-related neurodegenerative diseases (de Freitas et al., 2020). At the molecular level, these positive effects have been related to bioenergetic challenges and to the activation of transcription factors that induce the expression of proteins strengthening neurons' resistance to metabolic, oxidative, excitotoxic, and proteotoxic stresses (Daniele et al., 2018). Moreover, several brain functions are mediated by epigenetic regulation of neural genes, and their dysregulations result in neuronal disorders (Bertogliat et al., 2020). This Research Topic provided overview of the current knowledge on the epigenetic and metabolic modifications in response to physical activity. In this special issue, papers examined the role of intracellular signals, cell metabolism, and epigenetics assisted with the beneficial effects of exercise on brain health.

Zhang et al. reported that voluntary wheel running exercise improved cognitive deficits and attenuated the Aβ deposits in the hippocampus of APP/PS1 Alzheimer's disease (AD) model transgenic mice. Importantly, the authors demonstrate that treadmill exercise results in modulating microglia-related neuroinflammation in the early stage of AD pathology progression. The PA significantly reduced the gene transcription of inflammatory cytokine tumor-necrosis factor alpha (TNF-α) and interleukin-1-beta (IL-1β) thereby attenuating the production and deposition of Ab, and cognitive impairment. These results deeply elucidate the mechanisms underlying the positive effects of PA on AD progression that has been widely reported in literature (Lin et al., 2015; Xiong et al., 2015). It has been widely accepted that different exercise regimes evoke several beneficial effects in various brain functions (Liu et al., 2019). Another pivotal parameter that affects the potential benefits of the PA is the exercise modes. In this sense, the practice of open-skill exercises (i.e., badminton, football, tennis) and closed-skill exercises (i.e., swimming, jogging, cycling,) has been associated with an improvement of working memories (Chen F-T. et al.). Interestingly, the open-skill PA caused an improvement of neural activation in the prefrontal and anterior cingulate cortex regions in middle-aged adults.

Exercise interventions have been shown to attenuate brain aging *via* restoring Wnt signaling and corresponding targets, including PI3K/Akt pathway (Chen D. et al.). Moreover, the signals related to insulin resistance has been demonstrated as an independent predictor of postoperative cognitive dysfunction in aging population who undergo surgery, and, in this view, exercise may be considered an effective intervention of patients at risk (He et al.).

Concerning the role of epigenetics, a contributing review has summarized the impact of exercise on cognitive function and brain health, through the modulation of DNA methylation, and has a great potential as a non-pharmaceutical intervention to mitigate brain decline in women with breast cancer (Wagner et al.). Another brief overview summarized the current understanding of mild cognitive impairment in Parkinson's Disease, by considering physical activity as a non-pharmacological intervention in neuroprotection (Cammisuli et al.).

To date, several reports provide data on the positive correlation of PA and the improvement of brain functions in the population. However, the molecular mechanisms and the epigenetic regulation involved in this action is still unclear and is a fascinating area of investigation for the scientific community.

## Author Contributions

All authors listed have made a substantial, direct and intellectual contribution to the work, and approved it for publication.

## Conflict of Interest

The authors declare that the research was conducted in the absence of any commercial or financial relationships that could be construed as a potential conflict of interest.
